# T cell receptor rearrangement excision circles (TREC) study as an approach to "in vivo" thymus gland function investigation

**DOI:** 10.1186/ar3638

**Published:** 2012-02-09

**Authors:** Natalia Lakhonina, Mark Goloviznin, Almira Donetskova, Margarita Nikonova, Alexander Yarilin, Yulia Buldakova, Anna Tektova

**Affiliations:** 1 Department of Internal Diseases of Dental faculty, Moscow State University of Medicine and Dentistry, Russia; 2 Laboratory of cell immunology, Research Center Institute of Immunology, Moscow, Russia

## 

Age-dependent changes in immune system such as thymus gland involution, T-cells amount decrease, are typical both for autoimmune diseases (Rheumatoid arthritis - RA), and progressive atherosclerosis characterized as "accelerated ageing". But till now processes of T-cell maturation were studied only by indirect methods. The introduction of T-cell receptor excision circle (TREC) PCR-assay seemed to enable direct detection of recent thymic emigrants in peripheral blood and therefore the quantification of thymic output [1]. High TREC levels were detected during childhood, and were decreasing with age, but TREC-expressing cells are not completely lost in the elderly. At the first stage of our investigation we studied TREC level in 3 groups of healthy donors: 16 people. 16 - 30 years old (group 1, TREC Median 0,156299 Units), 8 persons 30 - 45 years old (group 2, TREC Median 0,08782 Units) and 9 people over 45 years (group 3 TREC Median 0,051858 Units). Thereby we confirmed age-related decline of thymic output in healthy donors.

In RA patients we found age-dependent statistical definite difference of TREC expression. In the 1-st group (n-12, age range 40,4+2,8 y) TREC amount was following: Median 0,00766 I/U lower level 0,00045, upper level 0,01961. In the 2-nd group (n = 16, age range 57,5+1,32) TREC were diminished (Median 0,00065, lower level 0,000002, upper level 0,00095). Detected high TREC amount in some young RA patients is not entirely consistent with the data of literature.TREC level in patients with chronic forms of coronary heart disease (age 55 - 70 years old) was lower but comparable with donors group 3 (TREC Median 0,0200 Units). Unexpectedly high level of TREC comparable with donors group 2 we detected in patients with Acute Myocardial Infarction (AMI) (10 patients, age range 48 - 71 y) (TREC Median 0,089845 Units). According to our viewpoint, the content of TREC in peripheral blood lymphocytes depends both on thymic output and "peripheral" factors, such as survival time of "naive" T cells in periphery. Recent data give evidence that the up-regulation of Th1 cell-functions and interferon-γ hyperproduction existed in patients with AMI after the onset of symptoms. This may participate in the immune-mediated ventricular remodeling after AMI. The slowing of "naive"-T-cells turnover and Th1/Th2 imbalance could be the reason of TREC increase in AMI patients.

The work is done in framework of project 11-04-01670 sponsored by Russian Foundation of Basic Research. Project director Dr. Goloviznin M.V.
